# The detection accuracy of cone beam CT for osseous defects of the temporomandibular joint: a systematic review and meta-analysis

**DOI:** 10.1038/srep34714

**Published:** 2016-10-06

**Authors:** Ruo-han Ma, Shuang Yin, Gang Li

**Affiliations:** 1 Department of Oral and Maxillofacial Radiology, Peking University School and Hospital of Stomatology, Beijing, China

## Abstract

The purpose of this review was to evaluate whether cone-beam computed tomography (CBCT) is reliable for the detection of bone changes of the temporomandibular joint (TMJ). Studies collected from the PubMed, Web of Science, Cochrane Library, ScienceDirect, Embase, Wanfang and CNKI databases were searched, and the publishing time was limited from January 1990 to December 2015. Eight studies (23 experimental research groups) were eventually included for further analysis. The pooled sensitivity was 0.67 and the pooled specificity was 0.87, which leads to a relatively large area (0.84) under the Receiver Operating Characteristic (ROC) curve. The related pooled positive likelihood ratio (+LR) and the pooled negative likelihood ratio (−LR) were 5.2 and 0.38, respectively. The subgroup analysis was conducted for four subgroups categorized by voxel size (≤0.2; >0.2, ≤0.3; >0.3, ≤0.4; >0.4, and ≤0.5 (mm)), and the “>0.4, ≤0.5” subgroup had a higher pooled sensitivity and pooled specificity than the other groups. The present study demonstrates that CBCT has a relatively high diagnostic accuracy for TMJ bone changes, although its reliability is limited. Voxel size did not play a role in the accuracy of CBCT.

Temporomandibular joint disorders (TMD) is a collective term^1^, and these disorders can affect the osseous components of the temporomandibular joint (TMJ), the soft-tissue components of the TMJ and the related muscles^2^. Unlike other joints in the human body, the TMJ is covered by a layer of fibrocartilage and the mandibular condyles can be damaged due to cartilage degeneration. Additionally, the bone can also initiate arthritis because of the particular dynamics in the maxillofacial area^3^.

Generally, a clinical examination alone is insufficient to fully assess the osseous and soft tissue component changes of the TMJ, and imaging is useful for the diagnosis process^2^. For the diagnosis and treatment of TMD, many imaging modalities have been used to evaluate the morphological changes of the TMJ. For the observation of the soft tissue of the TMJ, magnetic resonance imaging (MRI) is considered the gold standard because of its excellent soft tissue contrast^4^. Computed tomography (CT) and cone-beam computed tomography (CBCT) are often used to diagnose the defects of hard tissues of the TMJ. Compared with conventional image modalities and CT, CBCT has several advantages in detecting the bone changes of the TMJ, such as it provides three-dimensional images similar to CT but offers a relatively low radiation dose and high spatial resolution images for hard-tissue structures^5^,^6^,^7^.

Many researchers have focused on the diagnostic accuracy of the bone changes of the mandibular condyle using CBCT and have concluded that it is a useful imaging modality for the detection of TMJ bone changes^8^,^9^,^10^,^11^. This conclusion, however, was drawn from an impression of the results presenting in the related studies rather than a meta-analysis of the synthesized data. The aim of the present study was thus to synthesize the data from the published studies and to assess the power of CBCT in diagnosing osseous changes of the TMJ.

## Methods

### Search criteria

Studies collected from the PubMed, Web of Science, Cochrane Library, ScienceDirect, Embase, Wanfang and CNKI databases were searched, and the time was limited from January 1990 to December 2015. There was no language restriction on the search. A hand search for relevant references and grey literature was also performed to identify studies missed in the electronic search. The search strategy for each data base or search engine is shown in Table 1.

### Study selection and data extraction

**Figure 1:**
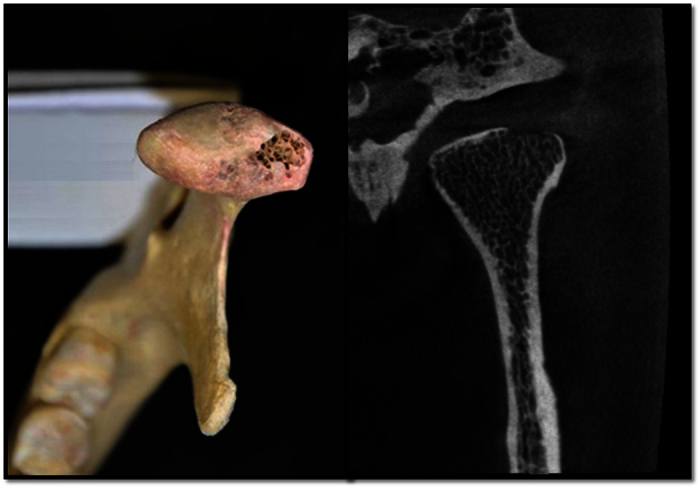
Human skull TMJs osseous defect sample and the corresponding CBCT images.

The whole search process followed the Preferred Reporting Items for Systematic Reviews and Meta-Analyses (PRISMA) statement^12^ and was carried out by two investigators (RHM and SY). These two individuals (RHM and SY) independently checked and removed duplicates and selected the included studies step by step. The investigators (1) sorted the studies on the basis of titles and abstracts, (2) selected the studies focusing on the diagnosis of bone changes of the mandibular condyle using CBCT by reading the articles, and (3) excluded the studies that met the exclusion criteria. Studies identified in this step were included in this study. Disagreements were resolved by discussion or were referred to experts. The study selection procedure was conducted in EndNote, version 17 (Thomson Research Soft, Stamford, CT, USA). The inclusion criteria were as follows: original *ex vivo* research, with the research subject being CBCT, and bone changes of the mandibular condyle. The exclusion criteria were as follows: animal trials, inappropriate literature type (case report, review or systematic review), the use of CBCT as a diagnostic gold standard, the use of cone-beam computed arthrotomography as the diagnostic method, and incomplete information about sensitivity/specificity or the cutoff value and information about the corresponding statistics was not sufficient to deduce sensitivity/specificity or the cutoff value. Figure 1 shows the dried human skull TMJ with bone defects and the corresponding CBCT images. Data information from each study such as sample size, types of bone change, the CBCT unit model, scanning parameters (tube voltage, tube current, scanning time, field of view and voxel size), the blinding method, gold standard, diagnostic scoring type and the message from the cross table of the diagnostic experiment were independently extracted and recorded by two investigators.

### Quality assessment and the risk of bias

Quality Assessment of Studies of Diagnostic Accuracy-2 (QUADAS-2)^13^ was used to assess the quality of the included studies. The assessments were estimated independently and checked by two investigators (RHM and SY). The quality assessment tool comprises the following four domains: sample selection, index test, reference standard and time and flow. Using the same standard for all samples, the risk of bias was evaluated by the following items: case-control design, inappropriate exclusion, blinded index test, pre-specified threshold, correct gold standard classification, blinded gold standard, a clear description of the gold standard, a proper interval between the test and standard, same standard for all samples. Each item was scored as “+” (yes), “−” (no) or “?” (unclear). For the evaluation of publication bias, the linear regression method was used to test the asymmetry of Deeks’ funnel plot. These procedures were performed in Review Manager, version 5.3 (The Nordic Cochrane Centre, The Cochrane Collaboration, Copenhagen, Denmark) and Stata, version 14.0 (Stata Corp LP, College Station, Texas, USA).

### Data synthesis and analysis

The true-positive, false-positive, false-negative, and true-negative values were calculated from the sensitivity and specificity values and were used to compute the pooled statistics. For the studies that provided the Receiver Operating Characteristic (ROC) curve only, the investigators extracted the points from the plots and computed the highest Youden’s index to find out the best cutoff value (the best sensitivity and specificity). The process was conducted using Get Data Graph Digitizer, version 2.26.0.20. The bivariate model (a random-effects model) was used to calculate the pooled sensitivity, the pooled specificity, the pooled positive likelihood ratio (+LR) and the pooled negative likelihood ratio (−LR). The +LR is the result of sensitivity/(1-specificity), and the −LR is the result of (1-sensitivity)/specificity. The Summary Receive Operating Characteristic (SROC) curve and the area under the curve (AUC) were drawn and computed if necessary. A heterogeneity test was carried out after data synthesis. A threshold analysis ensured non-heterogeneity of threshold effects, and thus other types of heterogeneity from non-threshold effects were examined. A meta-regression analysis or subgroup analysis was used to identify the sources of heterogeneity in terms of the number of influential factors. The computational process was carried out using Stata, version 14.0 (Stata Corp LP, USA) and Meta-Disc, version 1.4 (http://www.hrc.es/investigacion/metadis.html).

## Results

**Figure 2:**
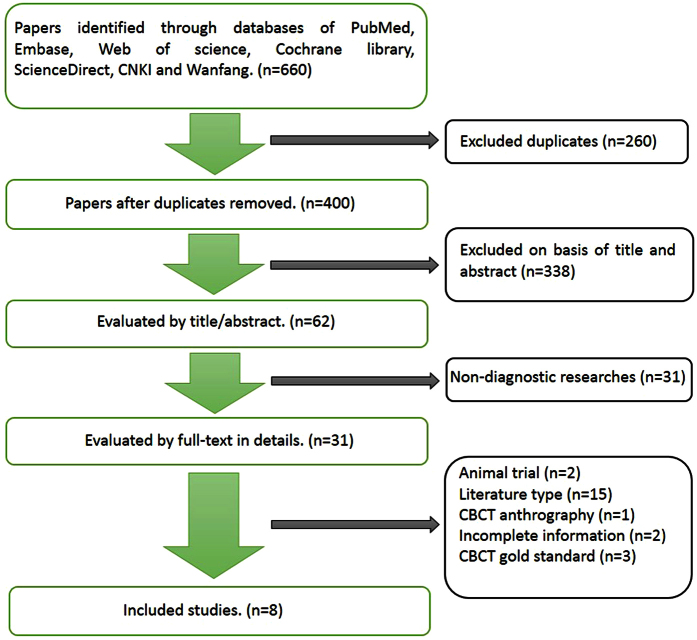
Flow chart.

In the aggregate, 660 studies were identified on the basis of the search criteria, and 400 publications remained. Based on the title and abstract, 338 studies were excluded because their topics did not focus on CBCT and bony defects of the mandibular condyle. Thirty-one studies were eliminated from the remaining literature, due to the non-specification of the detection accuracy of bone changes. In the remaining 31 studies, 8 were finally selected in accordance with the inclusion and exclusion criteria (Fig. 2).

**Figure 3:**
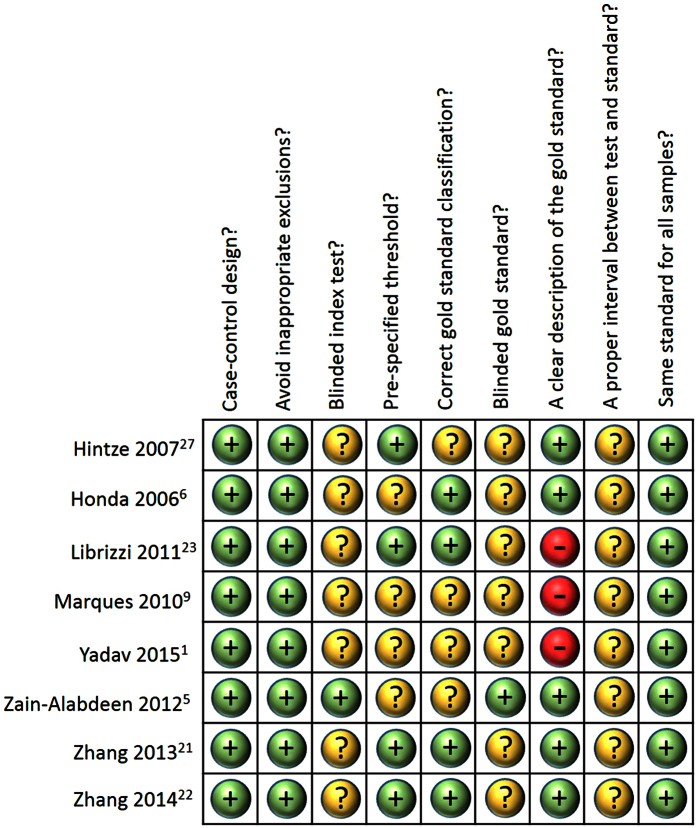
Risk of bias evaluation of the methodological quality of the included studies.

The meta-analyses were subsequently conducted on the eight studies (23 experimental groups). A total of 976 observed temporomandibular joint sites were studied and 478 of the sites had bone changes. The overall methodological quality of the studies is shown in Fig. 3.

### Publication bias

**Figure 4:**
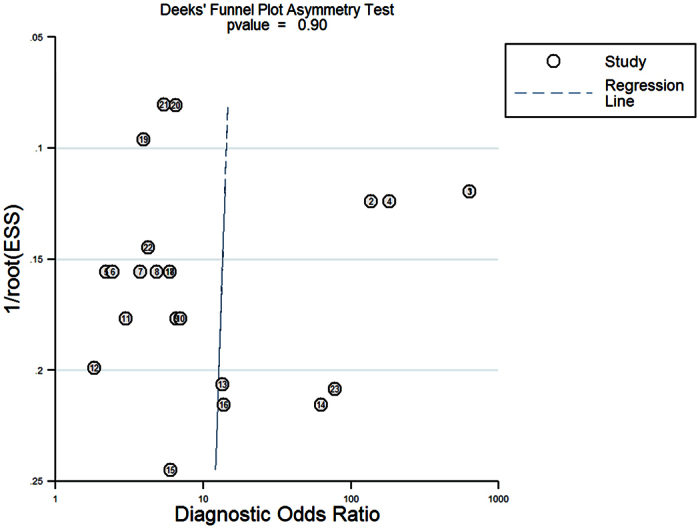
Deeks’ funnel plot.

The statistical results showed that the P-value of the linear regression test was 0.90 and the regression line for Deeks’ funnel plot was nearly at a ninety degree angle to the Axis diagnostic odds ratio (Fig. 4).

### Meta-analysis

**Figure 5:**
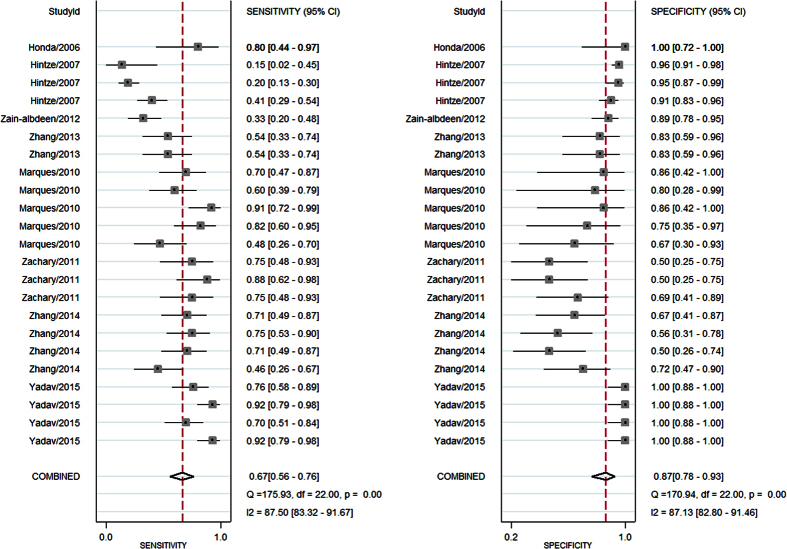
Forest plot of the included studies.

**Figure 6:**
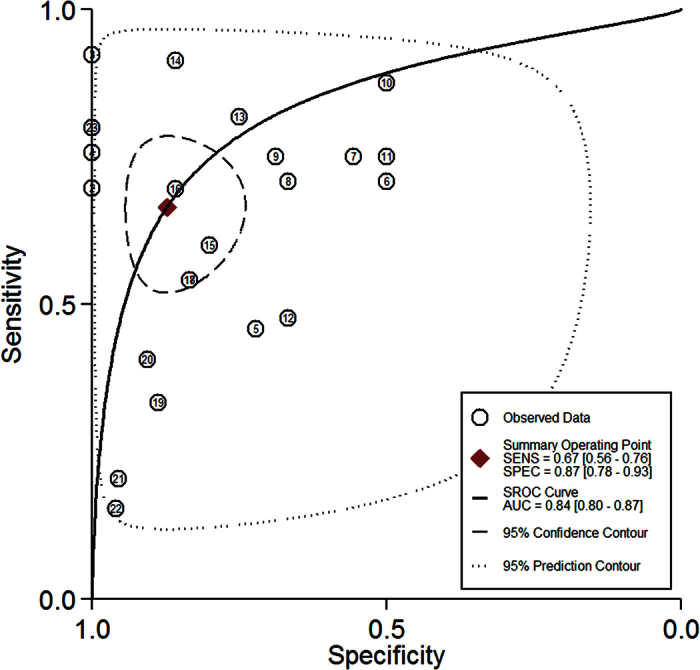
Summary receive operating characteristic curves.

Figure 5 presents the forest plots of sensitivity and specificity for all experimental research groups from each study. The pooled sensitivity and specificity and the I^2^ value, which describes the heterogeneity of the non-threshold effect and is a percentage of non-threshold variation in the total variations among studies, are also shown in Fig. 5. For all of the included studies, the pooled sensitivity was 0.67 (95% confidence interval (95%CI): 0.56 to 0.75) and the pooled specificity was 0.87 (95%CI: 0.78 to 0.93). The SROC curve was drawn to express the general diagnostic accuracy, and the AUC was 0.84 with 95%CI from 0.80 to 0.87 (Fig. 6). For all of the included studies, the pooled +LR was 5.2 (95%CI: 2.9 to 9.4) and the pooled −LR was 0.38 (95%CI: 0.28 to 0.52).

According to the threshold analysis result, the Spearman correlation coefficient was 0.052, with a corresponding P-value of 0.813. The I^2^ values of the pooled sensitivity and specificity were 87.50% and 87.13%, respectively. Voxel size was the only collected influencing factor, so the source of heterogeneity was analyzed accordingly. The included studies were grouped into four categories according to the voxel size of the experimental CBCT units. The pooled sensitivity, the pooled specificity and the corresponding I^2^ values were computed and the results of the subgroup analysis are shown in Table 2.

## Discussion

In the present study, *in vivo* studies were excluded from the meta-analysis. This is because in *in vivo* studies, it is difficult to know the corresponding reality of bone changes if a real patient is examined, and thus, there is no gold standard for reference. This directly affects the calculations of the sensitivity, specificity and ROC curve^14^,^15^,^16^,^17^, which are essential for a meta-analysis.

The QUADAS-2 was introduced to estimate the quality of the included studies. According to the theme of the present review, the investigators specified nine items to assess the quality of the included studies. To determine the true positive and true negative numbers of the studied objects, gold-standard was an important factor. Among the studies, three articles did not provide how the gold-standard or a reference standard were determined while the others give a gold-standard determined by naked eye with or without help of magnification glasses. Besides, only one study applied the blinding method to the research process.

One criticism regarding the validity of systematic reviews and meta-analyses is publication bias^18^, which is a widely known phenomenon existing in the clinical medical literature. Studies with positive results have more opportunities to be published than studies with negative results^19^. If there is a publication bias among the included studies, the combined results are misleading^20^. To prevent such a bias, a linear regression analysis was conducted and the P-value was 0.861, which represents no obvious asymmetry of the funnel plot. An approximately 90-degree angle was found between the regression line and diagnostic odds ratio in Deeks’ funnel plot, which also indicates that the possibility of publication bias among the included studies is rather small.

Usually diagnostic accuracy is represented by the area under a ROC curve. The curve is plotted according to a five-point rank scale assessment in imaging diagnosis. Cutoff points on the ROC curve correspond to different sensitivity and specificity levels. The best cutoff value represents the optimal point with the highest sensitivity and specificity. To conduct a meta-analysis on diagnostic accuracy, values of sensitivity and specificity should be known. Thus, for those studies that did not provide sensitivity and specificity values, they were extracted from the ROC curves. In the present study, the investigators computed the Youden’s index to determine the best cutoff value from which to extract the corresponding sensitivity and specificity for the included studies^21^,^22^,^23^. In this way, the pooled sensitivity (0.67) and the pooled specificity (0.87) were obtained and led to a relatively large AUC of 0.84 (Fig. 6). This result demonstrates a relatively high diagnostic accuracy of TMJ bony defects. This result is in agreement with the results from most of the previous studies in which CBCT is considered to be a dose-effective modality with high imaging resolution when compared to multi-slice computed tomography and traditional radiography (e.g., panoramic tomography)^6^,^7^,^11^,^24^,^25^. However, the pooled sensitivity is not as good as the specificity.

**Figure 7:**
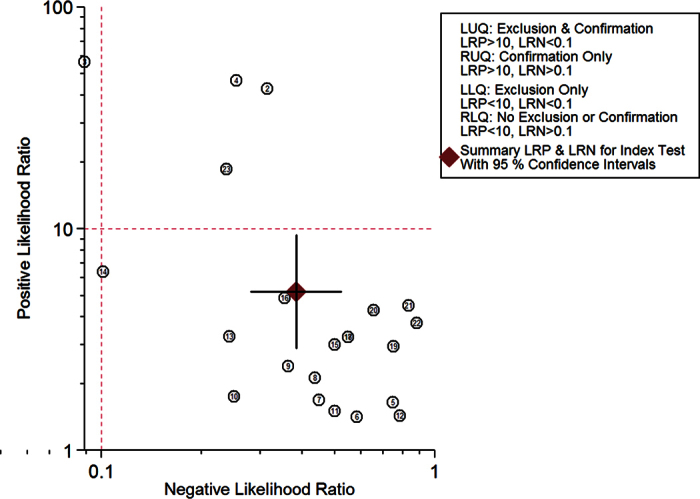
Likelihood ratio scatter gram.

The +LR is the sensitivity/(1-specificity), and the −LR is the (1-sensitivity)/specificity. It is generally acknowledged that if the +LR>10, the potential of the method used to correctly diagnose the studied disease is increased, and when −LR＜0.1, the possibility of correctly excluding the studied disease increases. If the LR equals 1, the method has no diagnostic value^26^. In the present study, the corresponding pooled +LR and the pooled −LR were 5.2 and 0.38, respectively. These values are not within the range of the aforementioned threshold values that are representative of a reliable diagnostic value. This is also illustrated in the likelihood ratio scatter-gram where the summary +LR and −LR values are in the right lower quarter of the coordinate system and only one experimental group was in the left upper quarter (Fig. 7).

The absence of heterogeneity of the threshold effect led to the analysis and discussion of the heterogeneity of a non-threshold effect. According to our results, the Spearman correlation coefficient of all the experimental groups was 0.052 and the corresponding P-value was 0.813, which indicate that no threshold effect existed in the included 8 studies. The I^2^ value describes the heterogeneity of the non-threshold effect, which is the percentage of non-threshold variation of the total variation among studies^26^. The I^2^ from the present study was 87.50 for the pooled sensitivity and 87.13 for the pooled specificity. These values are relatively high and indicate heterogeneity among the studies. To identify possible influencing factors, further analyses are needed. However, except for voxel size, no other information, such as the scanned field of view, the size of the bone defect and the CBCT unit were provided by all of the included studies. After analyzing the effect of voxel size, which was provided by most of the studies (except for study of Hintze *et al*.^27^), the heterogeneity values decreased by varying degrees, and in several subgroups, the heterogeneity decreased to 0.0%. These observations indicate that the voxel size is one of the sources of non-threshold heterogeneity. It is notable that the pooled sensitivity and the pooled specificity of the “>0.4, ≤0.5” voxel subgroup were higher than that those of the other three subgroups. Thus, there is no relationship between voxel size and diagnostic accuracy, which is similar to the result from the study by Lukat 2015^14^.

## Conclusions

CBCT has a relatively high diagnostic accuracy for the detection of TMJ bone changes. However, the reliability of the diagnostic accuracy is limited because the +LR/−LR values are not within the ideal threshold range. Voxel size may have no effect on diagnostic efficacy. To improve the clinical diagnosis accuracy and the clinical application of CBCT, more normative studies and further research are needed.

## Additional Information

**How to cite this article**: Ma, R.-h. *et al*. The detection accuracy of cone beam CT for osseous defects of the temporomandibular joint: a systematic review and meta-analysis. *Sci. Rep.* **6**, 34714; doi: 10.1038/srep34714 (2016).

## Figures and Tables

**Table 1:** Search strategy.

Database	Search strategy
PubMed (Medline)	All field: (“Spiral Cone-Beam Computed Tomography”[Mesh] or “Cone-Beam Computed Tomography”[Mesh] or “digital volume tomography” or “digital volumetric tomography” or “cone-beam ct” or cbct) AND (“Temporomandibular Joint Disorders”[Mesh] or “Temporomandibular Joint Dysfunction Syndrome”[Mesh] or tmd or “temporomandibular joint osteoarthriti*” or “temporomandibular joint osteoarthrosis” or “temporomandibular joint arthritis” or “tmj erosion” or “tmj inflammation” or “bony defect*”)
Web of Science	**TOPIC:** (“digital volume tomography” or “digital volumetric tomography” or “cone-beam computed tomography” or “cone-beam ct” or cbct) *AND* **TOPIC:**(“temporomandibular joint disorders” or “temporomandibular joint osteoarthriti*” or “temporomandibular joint osteoarthrosis” or “temporomandibular joint arthritis” or “tmj erosion” or “tmj inflammation” or “bony defect*”)
	**Timespan:** 1990–2015.
Cochrane Library	Search all text: (“digital volume tomography” or “digital volumetric tomography” or “cone-beam computed tomography” or “cone-beam ct” or cbct) AND Search all text: (“temporomandibular joint disorders” or “temporomandibular joint osteoarthriti*” or “temporomandibular joint osteoarthrosis” or “temporomandibular joint arthritis” or “tmj erosion” or “tmj inflammation” or “bony defect*”)
ScienceDirect	(“digital volume tomography” or “digital volumetric tomography” or “cone-beam computed tomography” or “cone-beam ct” or cbct) and (“temporomandibular joint disorders” or “temporomandibular joint osteoarthriti*” or “temporomandibular joint osteoarthrosis” or “temporomandibular joint arthritis” or “tmj erosion” or “tmj inflammation” or “bony defect*”)
Embase	(“digital volume tomography” or “digital volumetric tomography” or “cone-beam computed tomography” or “cone-beam ct” or cbct) and (“temporomandibular joint disorders” or “temporomandibular joint osteoarthritis” or “temporomandibular joint osteoarthrosis” or “temporomandibular joint arthritis” or “tmj erosion” or “tmj inflammation” or “bony defect”)
CNKI	Subject = cone beam CT or subject = CBCT and subject = temporomandibular joint disorders or subject = temporomandibular joint osteoarthrosis (in Chinese)
Wanfang	Subject: (cone beam CT or CBCT) and Subject: (temporomandibular joint disorders or temporomandibular joint osteoarthrosis) (in Chinese)

**Table 2:** Statistical results of subgroup analysis.

Voxel size	Pooled sensitivity	I^2^ value (%)	Pooled specificity	I^2^ value (%)
≤0.2	0.73	0.0	0.68	72.7
>0.2, ≤0.3	0.60	82.7	0.79	43.2
>0.3, ≤0.4	0.64	28.1	0.69	61.5
>0.4, ≤0.5	0.83	71.2	1.000	0.0
